# Does the Topology of Polymer Brushes Determine Their (Vapor-)Solvation?

**DOI:** 10.1021/acsmacrolett.5c00153

**Published:** 2025-05-28

**Authors:** Huaisong Yong, Jacco H. Snoeijer, Sissi de Beer

**Affiliations:** † Department of Molecules & Materials, MESA+ Institute, Faculty of Science and Technology, 3230 University of Twente, P.O. Box 217, 7500 AE Enschede, The Netherlands; ‡ Institute Theory of Polymers, Leibniz-Institut für Polymerforschung Dresden e.V., D-01069 Dresden, Germany; ¶ Physics of Fluids group, Faculty of Science and Technology, 3230 University of Twente, P.O. Box 217, 7500 AE Enschede, The Netherlands

## Abstract

When the topology of polymer brushes is changed from linear to cyclic or looped, many of the brush properties will be improved. Yet, whether such a topology variation also affects the (vapor-)­solvation and swelling of brushes has remained unclear. In fact, in a recent publication, Vagias and co-workers (*Macromolecular Rapid Communications* **2023**, *44* (9), 2300035) reported an unequal swelling for linear and cyclic brushes and challenged theoreticians to develop a new Flory–Huggins theory that includes topology effects. In this letter, we address this challenge and employ molecular dynamics simulations to study the vapor swelling of linear, looped, and cyclic brushes. We find that the emergence of equal or unequal swelling for different topologies depends on the definition of the grafting density that is kept constant in the comparison. When suitably defined, the degree of swelling is independent of the topology, and the Flory–Huggins theory for brushes will describe brush swelling for all topologies in the present study.

## Graphical abstract

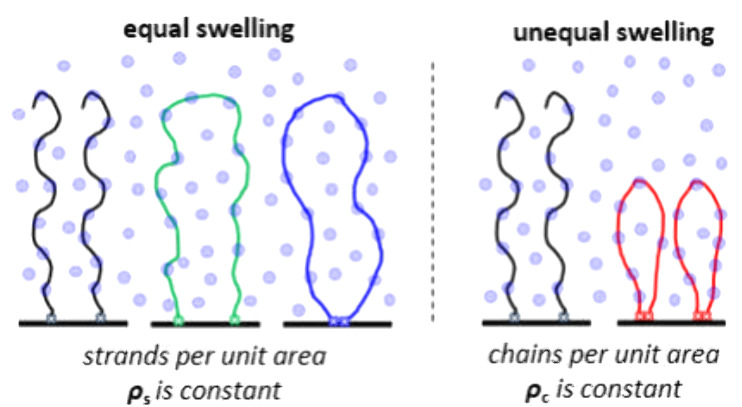

Polymer brushes consist of densely end-anchored polymers. These brushes have been studied extensively because of their broad range of potential applications, varying from biomedical systems to sensors and separation technologies.^1–3^ Approximately a decade ago, it was realized that changing the topology of polymers in brushes from linear to a cyclic or loop architecture can alter the performance of polymer brushes.^4,5^ For example, it has been reported that cyclic and looped polymer brushes foul less,^6–9^ have superior lubricating^10,11^ and self-cleaning^12,13^ properties, can improve colloidal stability,^14^ and respond more strongly to external stimuli^15^ than their linear counterparts.

To alter the topology and form loop or cyclic polymer brushes, various synthetic procedures can be followed:^16^ One can induce a reaction between the chain ends of linear polymers of degree of polymerization *N* and form loops of 2*N*,^17^ as depicted in Figure 1(a) in green. Alternatively, one can form loops by synthesizing α,ω-telechelic polymers and graft them by their functional end-groups on substrates.^18,19^ To form cyclic brushes, one can synthesize cyclic polymers in solution and then graft them to a substrate,^20^ as visualized in Figure 1(a) in blue and Figure 1(b) in red.

**Figure 1:**
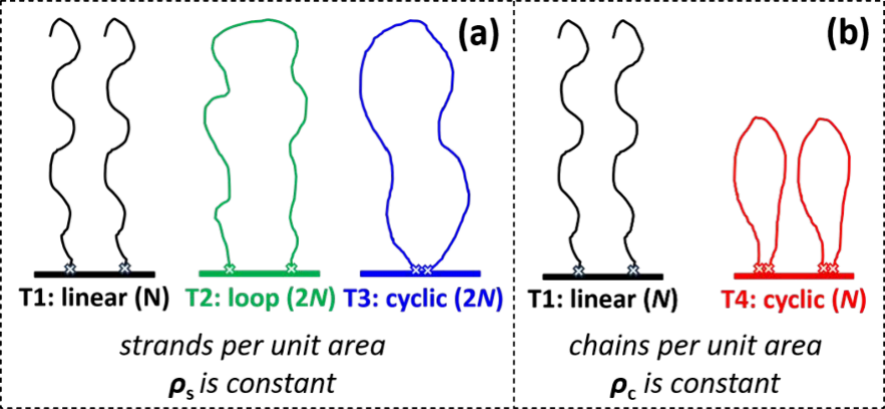
Different polymer brush topologies, as they are often studied in experiments and simulation. We compare two situations. (a) Topologies 1–3 are linear (T1), loop (T2), and cyclic (T3) brushes with polymers of length *N*, 2*N*, and 2*N*, respectively, at a constant number of surface strands per unit area ρ_s_, such that the number of monomers per unit area is constant. Consequently, the number of chains per unit area $\rho_{c}=\frac{1}{2}\rho_{s}$ for T2 and T3. (b) Topologies 1 and 4 are linear (T1) and cyclic (T4) brushes with polymers of length *N* and at a constant number of chains per unit area ρ_c_. For T4, it holds that ρ_s_ = 2ρ_c_.

When studying topology effects on the properties of polymer brushes, one has to consider several aspects, namely, the architecture (linear, loop, cyclic), the grafting density, and the chain length. The persistence length and branching functionality could have an effect as well,^21^ but they are not considered in the present work. Figure 1(a) depicts a topology variation, where the total number of monomers per unit area is kept constant. Whether these systems have the same or a different grafting density depends on the definition of the grafting density that is used. In the following, we will consider cyclic chains to have two chain ends anchored at the surface. We are aware that in experiments cyclic chains often have a single surface bond.^20,22^ Yet, we will show later that the actual bonding of cyclic chains becomes irrelevant in the current context. For the topologies in Figure 1a, the grafting density is constant when it is defined as the number of surface strands per unit area ρ_s_. However, when the grafting density is defined as the number of chains per unit area, ρ_c_, the grafting density for the loop (T2) and cyclic (T3) brushes is half of that of the linear brushes (T1). The number of monomers per chain is increased from *N* for linear chains to 2*N* for looped and cyclic brushes to keep the amount of monomers per unit area constant. The topology variations, as sketched in Figure 1(a), are often studied in theory and simulations. These studies have shown that these topology variations have only a minor effect on the density distributions,^23,24^ swelling of the brush in liquid,^25–28^ and the mushroom-to-brush transition.^29^ The reason for this is that the polymers are stretched similarly under these conditions. Nevertheless, the lubricating properties,^30,31^ nonwettability,^32^ and antifouling performance^33^ are still predicted to be improved for cyclic/loop brushes compared to linear brushes due to the absence of free chain ends.^34^

In experiments, however, the topology variation is often as sketched in Figure 1(b).^6,9,10,20,35^ In these systems, chain length *N* and number of chains per unit area ρ_c_ remain constant. When considering that cyclic polymers can be seen as having two strands rising from the substrate, the density of strands at the surface ρ_s_ is twice as high for the cyclic brushes compared to linear brushes. Therefore, polymers in cyclic brushes experience a higher monomer density and excluded volume interaction, such that they will stretch more compared to linear brushes at the same ρ_c_.^36^ This will affect brush swelling and other properties, as well as its performance.^22^

In a recent publication, Vagias et al.^37^ used time-of-flight neutron reflectometry measurements to compare the swelling of linear and cyclic brushes upon exposure to water vapor. Their topology was varied, as sketched in Figure 1(b). They reported that the brush topology influences the swelling ratio of vapor-solvated brushes and concluded that this difference in swelling asks for a new Flory–Huggins-type theory that includes topology effects. They challenged theoreticians to develop such a new theory. The necessity for a new theory came as an interesting surprise because previous simulation work has shown that brush swelling *in liquid* has been found to be almost independent of the topology upon varying the topology as depicted in Figure 1(a). Thus, it requires simulations of brushes of different topologies *in vapor* specifically to further study this discrepancy.

Indeed, it has been known for linear brushes that vapor swelling is qualitatively different from swelling of brushes in a liquid: The swelling ratio (swollen brush height normalized by the dry brush height) depends on the relative vapor pressure,^38–40^ which is determined by the polymer solvent affinity^38,39,41^ and the grafting density.^42,43^ Furthermore, an adsorption layer is formed at the brush–air interface,^43^ and surface tension effects give rise to a sharper brush density decay than observed in liquid-swollen brushes.^44^ In previous theory and simulation articles, we have shown that the swelling of linear brushes can be modeled by the Flory–Huggins theory, when the entropic penalty for polymer stretching is incorporated.^43,45,46^ This raises the question of whether this model can be employed for looped or cyclic brushes as well.

In this letter, we present molecular dynamics simulations of *vapor*-solvated brushes that have different topologies, as presented in Figure 1(a) and (b). We study how the topology variations affect the swelling and partitioning of the solvent in the brushes. Moreover, we evaluate the necessity for a new Flory–Huggins-type theory to describe vapor solvation of brushes of different topologies.

To study the solvation of the brushes, we use coarse-grained molecular dynamics simulations, where the polymers in our brushes are described with the Kremer–Grest model.^47^ This model is known to qualitatively describe the static and dynamic properties of polymer brushes.^40,48^ The chemical potential of the vapor μ (and thereby the vapor pressure *p*/*p*_sat_) is kept constant using the grand canonical Monte Carlo (GCMC) procedure, as implemented in LAMMPS.^49^ The volume *V* of our simulation box and the temperature *T* are kept constant as well, such that we work in the μ*VT* ensemble. We describe this procedure in more detail in a previous article.^43^ The temperature is kept constant at *T* = 0.85ϵ/*k*_B_ (*k*_B_ being the Boltzmann constant) to ensure liquid–vapor coexistence.^43^ More details on the simulation parameters and procedures can be found in the Supporting Information (SI section A).

In the following, we use the reduced units derived from the Lennard-Jones (LJ) potential, with units of length σ and energy ϵ representing the zero-crossing distance and potential well depth, respectively. Typical values for these parameters are ϵ = 30 meV and σ = 0.5 nm.^50^ The default values of ϵ are all set to 1: ϵ_pp_ = ϵ_ss_ = ϵ_ps_ = 1ϵ. Therefore, the Flory–Huggins parameter χ = 0. We have simulated four different topologies, as shown in Figure 1:

**Topology 1 (T1)** consists of linear brushes of *N* = 100 beads (one bead represents approximately 3–5 monomers).^47^ We vary the chain grafting density between ρ_c_ = 0.1 and 0.6σ^–2^. These grafting densities translate to approximately 0.4–2.4 chains per nm^2^, which is experimentally achievable.^51^ For linear brushes, ρ_c_ is the same as the number of surface strands per unit area ρ_s_ and thus also ρ_s_ = 0.1–0.6σ^–2^.
**Topology 2 (T2)** consists of looped brushes of 2*N* = 200 beads. The number of surface strands per unit area is the same as in Topology 1 (ρ_s_ = 0.1–0.6σ^–2^). Consequently, ρ_c_ is half of ρ_s_ $\left(\rho_{c}=\frac{1}{2}\rho_{s}=0.05\text{−}0.3\sigma^{-2}\right)$ .
**Topology 3 (T3)** consists of cyclic brushes of 2*N* = 200 beads. Also, here the number of surface strands per unit area is the same as in Topology 1 (ρ_s_ = 0.1–0.6σ^–2^). Consequently, ρ_c_ is half of ρ_s_ $\left(\rho_{c}=\frac{1}{2}\rho_{s}=0.05\text{−}0.3\sigma^{-2}\right)$ .
**Topology 4 (T4)** consists of cyclic brushes of *N* = 100 beads. Here, the chain grafting density ρ_c_ is kept the same as for the linear polymer brushes in Topology 1 and varied between ρ_c_ = 0.05 and 0.3σ^–2^). A grafting density of ρ_c_ = 0.6σ^–2^ gave rise to too dense brushes. The number of strands per unit area ρ_s_ = 2ρ_c_ = 0.1–0.6σ^–2^.

We first examine polymer and solvent partitioning in the polymer brushes for the three topologies T1, T2, and T3 in Figure 1(a), where we keep the density for the surface strands constant (ρ_s_ = 0.3σ^–2^). The brushes are exposed to a solvent vapor at μ = −3.5ϵ, which translates to a relative vapor pressure of *p*/*p*_sat_ = 0.75. Figure 2(a) gives the equilibrium number density profiles for polymer beads (solid lines) and solvent (vapor) particles (circles and dashed lines) as a function of the distance from the substrate *z*, with the wall at *z* = 0. The oscillations near the wall are layering effects due to a wall symmetry breaking effect and can be ignored.

**Figure 2:**
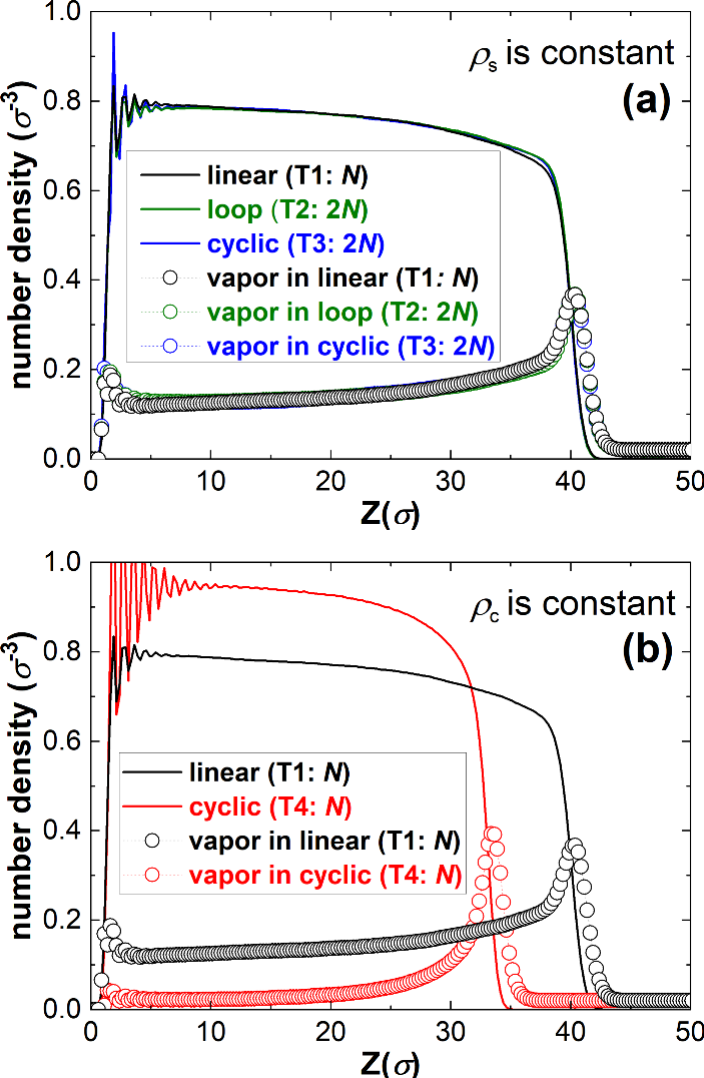
Polymer (solid lines) and solvent vapor (dashed lines, circles) density profiles. (a) Topology variations, where the total number of monomers per unit area and ρ_s_ is constant (ρ_s_ = 0.3σ^–2^, for brushes with linear polymers of *N* = 100 beads and ρ_s_ = ρ_c_ = 0.3σ^–2^ (T1, black), looped polymers of *N* = 200 beads and ρ_s_ = 2ρ_c_ = 0.3σ^–2^ (T2, green), and cyclic polymers of *N* = 200 beads and ρ_s_ = 2ρ_c_ = 0.3σ^–2^ (T3, blue); see also Figure 1(a)). (b) Density profiles for topology variations, where the chain length (*N* = 100) and ρ_c_ are constant (ρ_c_ = 0.3σ^–2^, for linear brushes of ρ_c_ = ρ_s_ = 0.3σ^–2^ (T1, black) and cyclic brushes of $\rho_{c}=\frac{1}{2}\rho_{s}=0.3\sigma^{-2}$ (T4, red) brushes; see also Figure 1(b)).

The three polymer density profiles in Figure 2(a) (solid lines) are approximately the same. They all show a sharper density decay at the brush–vapor interface (near *z* = 40σ) than the well-known gradual decay of brushes in liquid, as has been reported in previous experiments^44^ and simulations.^43,46^ There are only very small differences between the density profiles at distances between *z* = 35σ and *z* = 40σ. There, the polymer density of the looped and cyclic brushes is around 2% higher compared with the linear brushes. This difference is statistically significant and remains upon varying the initial condition and brush properties. For example, the difference increases for shorter chains (*N* = 50) to 4% or by lowering the grafting density to ρ_s_ = 0.1σ^–2^ to 3% (see SI section B, Figures S2–S4). We attribute the difference to the slightly higher translational entropy of the free chain ends for linear brushes. These observations are consistent with previous simulations in liquid, where also only negligible variations in the density profiles are observed when the topology is varied as in Figure 1(a).^23,24,33^

The dashed lines (circles) in Figure 2(a) give the density of the solvent vapor. The solvent partitioning for all three topologies is consistent with the distributions we previously observed for linear brushes under athermal condition (ϵ_ss_ = ϵ_pp_ = ϵ_ps_ = 1ϵ and thus χ = 0).^43^ In the bulk of the brush, the solvent number density increases only slightly from 0.12 to 0.2 over 5σ < *z* < 35σ. The solvent density goes through a maximum at the brush–vapor interface. The presence of an interface is energetically not favorable, and hence there will be an enrichment of the medium that will reduce the interfacial energy the most.^52–56^ For χ = 0, one can expect that the medium with the highest entropic gain (i.e., the solvent) will reside at the interface. Indeed, the density near the wall (*z* = 2) also shows a small maximum due to the interface formed there. We observe that between *z* = 35σ and *z* = 40σ the solvent density in the looped and cyclic brushes is slightly lower than that in the linear brushes, which is consistent with the small differences in the polymer density we described above. We thus conclude that for Topology 2 and 3, as defined in Figure 1(a), the absorption of vapor is nearly indistinguishable from that of the linear brush of Topology 1.

As mentioned earlier, we consider cyclic brushes to have two surface anchors, while Topology 3 in experimental systems often has one surface bond.^20,22^ To test if the number of surface bonds affects our results, we repeated the simulations and analysis for cyclic chains with single surface bonds. The density profiles of these brushes are indistinguishable from those presented in Figure 2 (see section C, Figures S5–7 in Supporting Information). Therefore, we conclude that the actual number of surface bonds is not important in the current context.

We now turn to the comparison with Topologies 1 and 4, as defined in Figure 1(b). This case corresponds to the comparison made by Vagias et al.^37^ For this topology variation, we observe clear differences between the linear (T1, black) and cyclic (T4, red) polymer brushes. The cyclic brushes absorb less solvent than the linear polymer brushes. The reason for this is that the cyclic polymers experience a higher density, which forces them to stretch more when dry already.^22^ When brush polymers are more stretched, the absorption of solvent will introduce a higher entropic penalty compared to brush polymers that are less stretched, such that swelling will be less. For the same reason, the swelling ratio also decreases upon increasing the grafting density for linear polymer brushes.^57,58^

The lower vapor solvent absorption in the cyclic brushes compared to the linear brushes shown in Figure 2(b) appears to be inconsistent with the conclusions from Vagias et al.^37^ who reported a higher swelling ratio for cyclic brushes compared to linear brushes. We do not have an explanation for this discrepancy. The authors propose that a new Flory–Huggins-type model is needed that incorporates topology effects to properly describe the swelling of cyclic brushes. However, we would like to argue based on our results in Figure 2(a) that this should not be necessary. When ρ_s_ is kept constant, solvent absorption is hardly affected by topology changes. Therefore, we anticipate that the Flory–Huggins theory for brushes in vapors^43^ can be used to describe the swelling of all topologies in Figure 1.

To verify that the swelling of brushes is indeed independent of the topology, provided that ρ_s_ is kept constant, we determine the swelling ratio *h*_swollen_/*h*_dry_ for the different topologies (T1–T4) and different grafting densities. The swelling ratio is calculated by determining *h*_swollen_ from simulations performed at μ = – 3.5ϵ (*p*/*p*_sat_ ≈ 0.75) and normalizing it by the dry height *h*_dry_ extracted from simulations without solvent vapor present. The brush heights *h*_swollen_ and *h*_dry_ are defined as the location of the inflection point in the polymer density (e.g., *h*_swollen_ = 39.9σ for the density profiles in Figure 2(a)). The density profiles for the swollen and dry brushes for the different grafting densities can be found in the Supporting Information. Figures 3(a) and (c) show *h*_swollen_/*h*_dry_ as a function of the number of chains per unit area ρ_c_ for different polymer–solvent interactions. At constant ρ_c_, the linear brushes (black squares) swell more than the looped (green triangles) or cyclic brushes (blue/red circles). As discussed above, this is expected because the looped and cyclic brushes experience a more crowded environment at a constant ρ_c_. Therefore, they stretch more under dry conditions already, such that the entropic penalty of further stretching is higher than for the linear brushes. When plotted as a function of ρ_s_, Figure 3(b) and (d) indeed show a collapse of all of the data for topologies T1–4. For a constant ρ_s_, the effective monomer density and excluded volume interactions experienced by the polymers are the same for each topology. Therefore, swelling is equal. We note that the swelling ratio is also independent of the chain length; the cyclic brushes of 2*N* (blue open circles) and *N* (red closed circles) give rise to the same swelling ratios as in Figure 3. The reason for this is that both *h*_dry_ and *h*_swollen_ have the same scaling relations and can be assumed to increase linearly with increasing *N*,^43,57^ so that the swelling ratio is independent of *N*.

**Figure 3:**
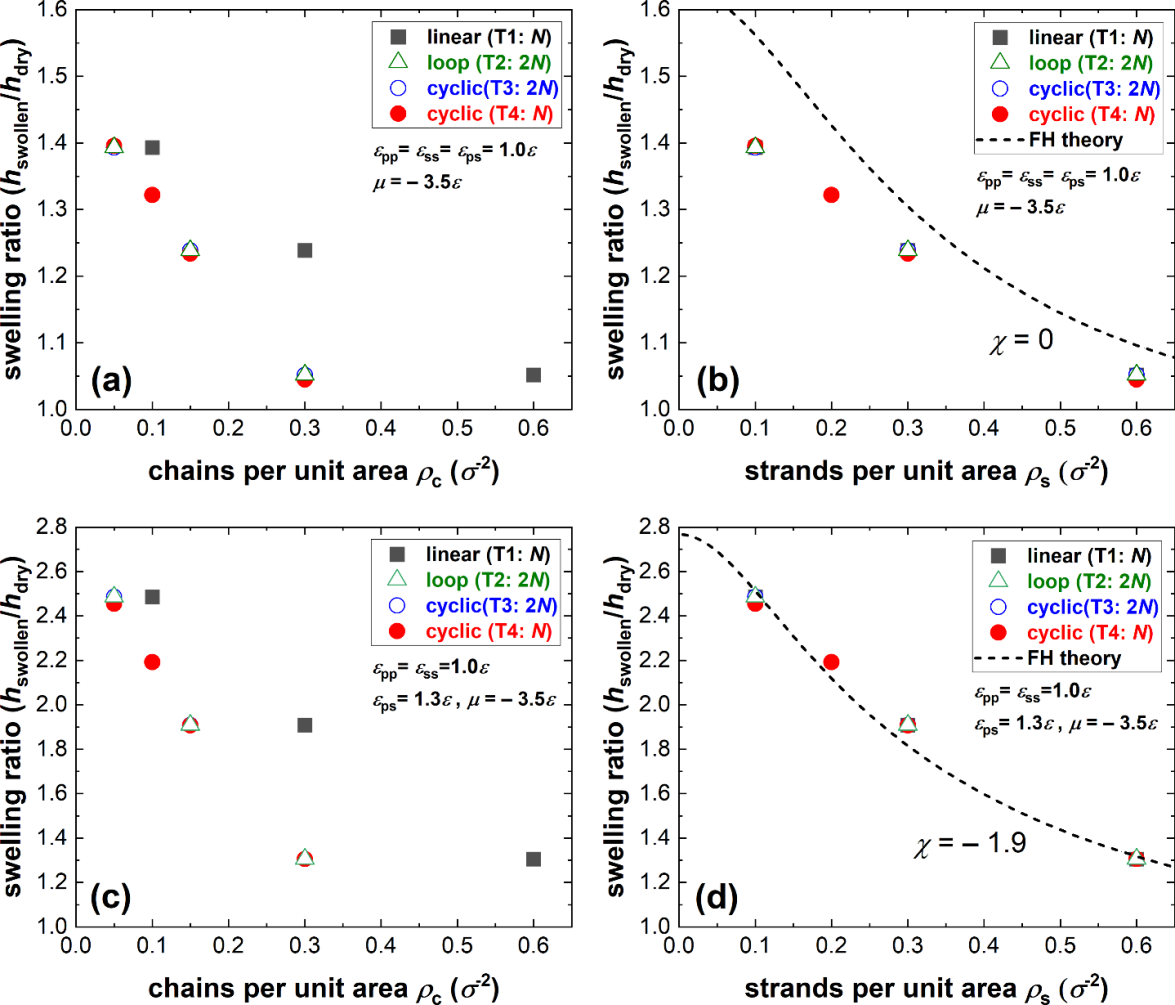
Swelling ratio *h*_swollen_/*h*_dry_ as a function of the grafting density for different topologies, namely, cyclic with polymer length 2*N* (T3 blue open circles), cyclic with polymer length *N* (T4, red circles), loop with length 2*N* (T2, green triangles), and linear with length *N* (T1, black squares), for different definitions of the grafting density and interaction parameters: (a) chains per unit area ρ_c_ (ϵ_ss_ = ϵ_pp_ = ϵ_ps_ = 1ϵ and thus χ = 0), (b) surface strands per unit area ρ_s_ (χ = 0), (c) chains per unit area ρ_c_ (χ = −1.9), and (d) surface strands per unit area ρ_s_ (χ = −1.9).

The observations described above have implications for the Flory–Huggins theory, as well. The equation describing vapor swelling of polymer brushes by the Flory–Huggins theory can be written as (for the derivation see SI section F):^40,43,45,56^

$$\ln\left(\frac{p}{p_{\mathrm{sat}}}\right)=\ln\left(1-\phi_{p}\right)+\phi_{p}+\chi \phi_{p}^{2}+\frac{3{\mathrm{\rho ̃}}^{2}}{\phi_{p}}$$ 1

In this equation, the polymer fraction in the brush ϕ_p_ is related to the solvent fraction in the brush by ϕ_s_ = 1 – ϕ_p_ and the swelling ratio by^40^

$$\frac{h_{\mathrm{swollen}}}{h_{\mathrm{dry}}}=\frac{1}{1-\phi_{s}}=\frac{1}{\phi_{p}}$$ 2

For linear brushes (T1), the grafting density ρ̃ in eq 1 can be defined as the number of chains per unit area ρ_c_ or the number of strands per unit area ρ_s_ alike. However, this equivalence breaks down for the looped and cyclic brushes in Topologies 2–4. For these topologies, the grafting density has to be defined as the number of strands per unit area (ρ̃ = ρ_s_) because the last term in eq 1 describes the entropic penalty for polymer stretching in the brush, which is constant for a constant ρ_s_, since the brushes experience the same monomer density. The fact that ρ_s_ is the relevant grafting density in the current context was already inferred from the collapse of data in Figure 3(b). The dashed lines in Figure 3(b) and (d) further compare the data quantitatively with the Flory–Huggins equation. As reported previously,^43^ we used a fitting routine to determine that χ = −1.9 for ϵ_ps_ = 1.3 (see SI section G). Despite the limitations of this mean-field theory, such as for example the Alexander–de Gennes assumption of a block-shaped density profile (SI section G for the comparison of the density profiles) and the assumption of Gaussian polymer stretching energy, it predicts brush swelling almost quantitatively. This is in agreement with other articles written on the topic.^40,43,46^ As reported in our previous work,^43^ near χ = 0 Flory–Huggins overestimates the swelling ratio by 5–20% (depending on ρ_s_). Yet, this overestimation is independent of the topology and does not change the conclusion that the Flory–Huggins equation can be used to describe the vapor solvation of brushes of all the different topologies T1–T4, when ρ̃ = ρ_s_.

In summary, we addressed the challenge posed by Vagias et al.^37^ and studied the vapor solvation of brushes of different topologies in chemical equilibrium with a vapor. We find that the vapor solvation of brushes is largely independent of the topology when the number of strands per unit area ρ_s_ is considered to be constant. When the number of chains per unit area ρ_c_ is constant, we observe that linear brushes swell more than looped or cyclic brushes. The reason for this is that for constant ρ_c_ looped or cyclic brushes are more crowded and stretched than linear brushes, such that it is energetically less favorable to absorb solvent. Our results indicate that the Flory–Huggins theory for linear brushes can be used to describe vapor swelling of the other topologies examined in this letter as well, provided that ρ_s_ is kept constant (ρ̃ = ρ_s_).

## Supplementary Material



## Notes

Detailed description of the Model and Methods used for the simulations and the density profiles used to extract the swelling ratios of Figure 3 (PDF)
