# Supplementary material for: Does the Topology of Polymer Brushes Determine Their (Vapor-)Solvation?
Source: ACS Macro Lett. 2025 May 28;14(6):816–21. doi: 10.1021/acsmacrolett.5c00153 (PMC12177944; doi:10.1021/acsmacrolett.5c00153)
Supplement: Supplementary file 1 [file mz5c00153_si_001.pdf]

Supporting Information *for*

"Does the Topology of Polymer Brushes Determine their (Vapor-)Solvation?"

Huaisong Yong[1,2], Jacco H. Snoeijer[3], and Sissi de Beer[1*]

[1]Department of Molecules & Materials, MESA+ Institute, University of Twente, 7500 AE Enschede, The Netherlands

[2]Institute Theory of Polymers, Leibniz-Institut für Polymerforschung Dresden e.V., D-01069 Dresden, Germany

[3]Physics of Fluids Group, Max Planck Center Twente for Complex Fluid Dynamics, Faculty of Science and Technology, University of Twente, 7500 AE Enschede, The Netherlands

[*]Correspondence author: Sissi de Beer (s.j.a.debeer@utwente.nl)

Section A: Simulation details

The general simulation setup and procedure are the same as those reported by our previous work **[1]**. In this work, as sketched in **Figure S1**, solvent partitioning is investigated using coarse-grained MD simulations of the brush–solvent system, alternated with grand-canonical Monte Carlo (GCMC, μVT ensemble) sweeps to maintain a constant solvent chemical potential (μ) and a constant temperature (T) in a region above the brush. Its implementation is based on a description according to Frenkel and Smit **[2]**. In this GCMC procedure, a set number of particle insertions and deletions are attempted and evaluated based on a Metropolis criterion. All simulations were performed using the MD package large-scale atomic/molecular massively parallel simulator (LAMMPS **[3]**).

Nonbonded pair-interactions in our simulation are described by a form of the well-known Lennard-Jones (LJ) potential:

$$U_{LJ}(r) = 4\varepsilon\left[\left(\frac{\sigma}{r}\right)^{12} - \left(\frac{\sigma}{r}\right)^{6}\right] \tag{1}$$

where r represents the distance between two particles, the depth of the potential well is given by ε, and σ is the size of a particle. The minimum occurs at $r_m = 2^{1/6}\sigma$. Specifically, the truncated and potential-shifted (SP) form of the Lennard-Jones potential is used:

$$U_{LJ,SP}(r) = \begin{cases} U_{LJ}(r) - U_{LJ}(r_c) & \text{for } r \le r_c \\ 0 & \text{for } r > r_c \end{cases} \tag{2}$$

where $r_c = 2.5\,\sigma$ is the cutoff distance for the interaction. This cutoff indicates that all interparticle interactions in our simulations are attractive at distances larger than 1.0σ. Throughout this work,

we use reduced Lennard-Jones units, meaning that ε and σ are used as energy and length units for our system, respectively. Consecutive beads along a polymer backbone are bonded via a finitely extensible nonlinear elastic (FENE) potential (**Equation(3)**) combined with a Weeks–Chandler–Anderson (WCA) potential (**Equation(4)**). The latter is equivalent to an LJ potential truncated at its minimum and shifted to zero at the cutoff, thereby making it purely repulsive. The total bonded potential is the sum of the FENE and WCA potentials (**Equation(5)**).

$$U_{FENE}\left(r\right)=-\frac{1}{2}KR_0^2\ln\left[1-\left(\frac{r}{R_0}\right)^2\right] \tag{3}$$

with

$$U_{WCA}\left(r\right)=\begin{cases}U_{LJ}\left(r\right)+\varepsilon & \text{for } r\leq 2^{1/6}\sigma\\ 0 & \text{for } r>2^{1/6}\sigma\end{cases} \tag{4}$$

and

$$U_{bond}\left(r\right)=U_{FENE}\left(r\right)+U_{WCA}\left(r\right) \tag{5}$$

In **Equation(3)**, R_0 is the maximum bond length and K is a spring constant. In our simulations, K is set to $30\varepsilon/\sigma^2$, R_0 is 1.5σ, and ε and σ are equal to 1.0. These parameters, borrowed directly from the Kremer–Grest model **[4]**, prevent bond crossing and other unphysical behaviors.

The entire system is thermostatted to a temperature of $T = 0.85\varepsilon/k_B$ using a chain of three Nosé–Hoover thermostats (which ensures proper sampling of the canonical ensemble **[5]**) with a damping constant of 0.15τ, where τ represents the reduced time unit derived from the Lennard-Jones potential, which is given by $\tau = (m\sigma^2/\varepsilon)^{1/2}$ where $m = 1.0$ is the mass of a bead in our simulations. k_B is the Boltzmann constant, which we take to be unity, as the energy scale of the simulations is arbitrary. The temperature of $T = 0.85\varepsilon/k_B$ was determined to allow vapor–liquid coexistence for the solvent. The GCMC chemostat is active every 10 000-time steps, where it attempts 1000 solvent particle insertions/deletions. These values were empirically determined to result in a good balance between simulation performance and convergence speed.

The polymer system is first equilibrated by running a short minimization using the conjugate gradient method, followed by running dynamics for 10 000-time steps with a limit imposed on the maximum movement of a particle in one time step of 1σ and a Langevin thermostat with a damping parameter of 1000τ. A second minimization is then performed. Finally, 200 000-time steps of more viscous Langevin dynamics are performed with a damping parameter of $100\ \tau$ and without the limit. This procedure is chosen to relax the system from the low-entropy initial state (fully extended chains) as quickly and efficiently as possible. After the equilibration, a production run is started in which solvent particles are introduced into the system by enabling the GCMC mechanism. The system is simulated for 60-million-time steps ($900\,000\tau$), as this ensures an

equilibrated state and adequate signal-to-noise ratio for all simulation cases. LAMMPS input files as well as a Python wrapper around LAMMPS are available online **[5]**.

For the equilibration, the LAMMPS default value for the time step (0.005τ) is used. For the production runs, the rRESPA multi-time-scale integrator **[6]** is employed with an outer time step of 0.015τ and a twofold shorter inner time step. This results in nonbonded pair interactions being computed every $0.015\ \tau$, but bonded interactions being computed every 0.0075τ.

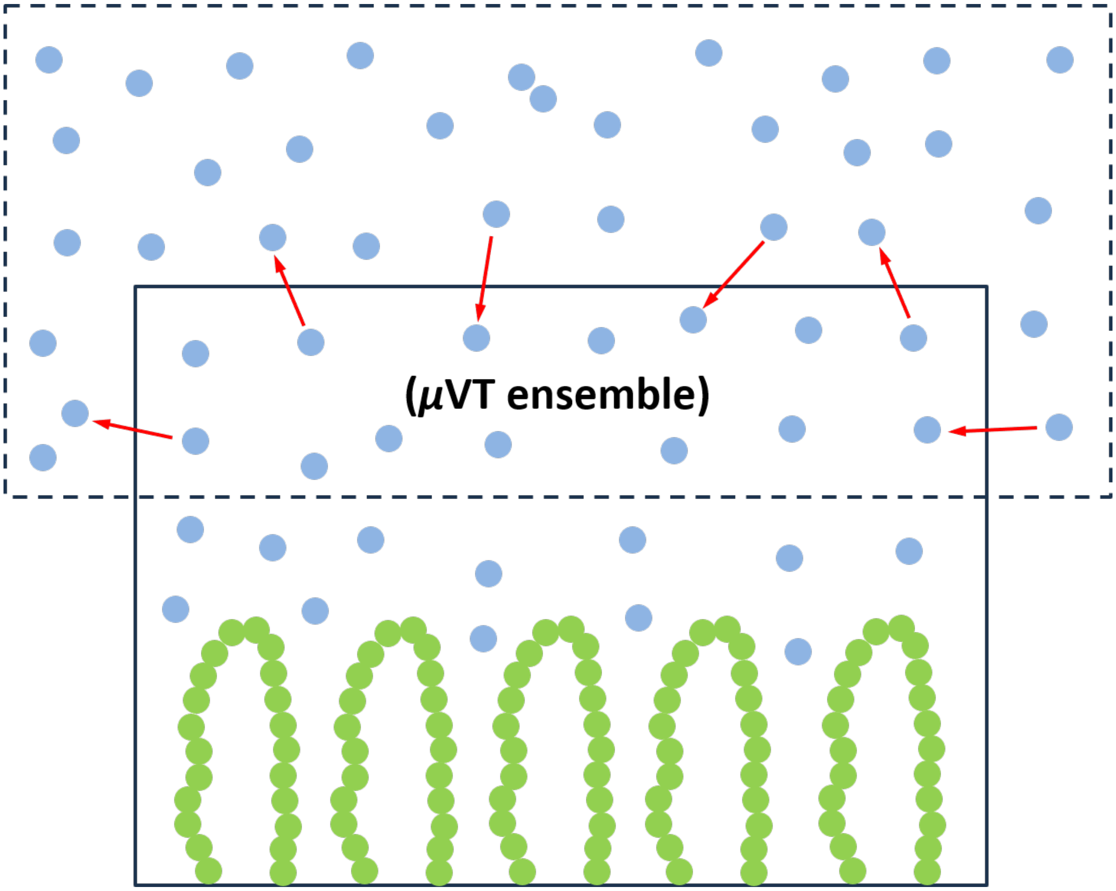

Figure S1. A graphical representation for loop polymer brushes (green filled circles) as an example in the GCMC-MD simulation box. The top part of the simulation box (indicated with solid borders) is in equilibrium with an implicit atmosphere (indicated with dashed borders). This equilibrium is maintained by a Metropolis algorithm that determines the success of insertion/deletion of a vapor particle (light blue filled circles) from the surrounding atmosphere into the simulation box as indicated by the arrows. Only the contents of the simulation box are explicitly simulated in the MD procedure between GCMC sweeps.

In this study a linear brush chain was modeled as N = 100 freely jointed bead spring chains, anchored at one end to an uncharged planar surface to form a regular 10 × 10 square grid. The linear chains were mono-disperse and modeled as N spherical beads with grafting density ρ_s = 0.1-0.6/σ^2 where σ is the diameter of a monomer. The lengths of loop and cyclic brushes were

modeled according to the grafting density and the chain length clarified by **Figure 1** of the main text. The rectangular simulation box with the fixed dimension of $V = L_x \times L_y \times L_z$ in the units of a monomer diameter (σ) had periodic boundaries in both (horizontal) x–y directions ($L_x = L_y = 10/\sqrt{\rho}$), while the (vertical) height was much larger than the contour chain length ($L_z = 157\sigma >> N\sigma$) and restricted by a wall to confine the vapor and monomer particles inside the simulation box. The vapor molecule was modeled as a single spherical bead with the same size of a monomer.

In this study to allow vapor–liquid coexistence for the solvent, we controlled the chemical potential of solvent (μ) rather than its relative vapor pressure (p/p_{sat}) at $\mu = -3.5\varepsilon$. In our simulations, the saturated vapor pressure (p_{sat}) is reached at about $\mu \approx -3.25\varepsilon$. This means that the relative pressures (p/p_{sat}) that corresponding to $\mu = -3.5\varepsilon$ is about 75%, which is definitely below vapor saturation and can be estimated by the numerical method reported by our previous work **[1]**. In order to compare the vapor-sorption characteristics of brushes with different topological structures (linear and cyclic/loop brushes), we used two interaction examples in our simulations for the interaction strength between monomer and monomer (ε_{pp}), between solvent and solvent (ε_{ss}), as well as between monomer and solvent (ε_{ps}). The first example is $\varepsilon_{pp} = \varepsilon_{ss} = \varepsilon_{ps} = 1.0\varepsilon$, the second example is $\varepsilon_{pp} = \varepsilon_{ss} = 1.0\varepsilon$ with $\varepsilon_{ps} = 1.3\varepsilon$.

The sorption behavior of the system is evaluated by analyzing density profiles of the polymer and the solvent over the z direction (averaged over x and y). During the simulation, these are dumped every 10 000-time steps (averaged over 100-time steps equally spaced out since the previous frame). To ensure properly equilibrated results, the first 95% of all frames are discarded and only the last 5% are time-averaged for further processing. For the calculation of several physical quantities, definitions of spatial limits are required. First, the brush height is defined by the inflection point (point of maximum slope, as determined using a Savitzky–Golay filter) in the polymer density profile. Second, we define an outer limit for the adsorption layer by an (arbitrary) lower threshold of $0.002/\sigma^4$ in the solvent density gradient. Any solvent beyond this point is considered vapor bulk. To mitigate discretization errors in the determination of the limits described above, the density profiles are spatially interpolated 10 times using a cubic spline interpolant prior to time-averaging. The amount of absorption (solvent inside the brush) is calculated as the integral of the solvent density profile up to the brush height, and similarly, the amount of adsorption is calculated as the integral of the solvent density profile from the brush height up to the adsorption layer end. The Python code that implements this procedure is available online **[7]**.

Section B: The chain-length and grafting density effects on the density profile

In this section, we show the chain-length and density effect on the density profile. In **Figure S2-S4**, we show the density profiles of polymer brushes with different topological variations, and related density profiles of solvent molecules in corresponding polymer brushes. In **Figure S2,** the chain length parameter N is 50, in other figures N is 100. We see that the general properties of density profiles do not change. For N is 50, the number density for the cyclic and looped brushes is around 4% higher than for the linear brushes, while for $\rho_s = 0.1\sigma^{-2}$ this difference is 3% and for $\rho_s = 0.6\sigma^{-2}$ this difference is <1%.

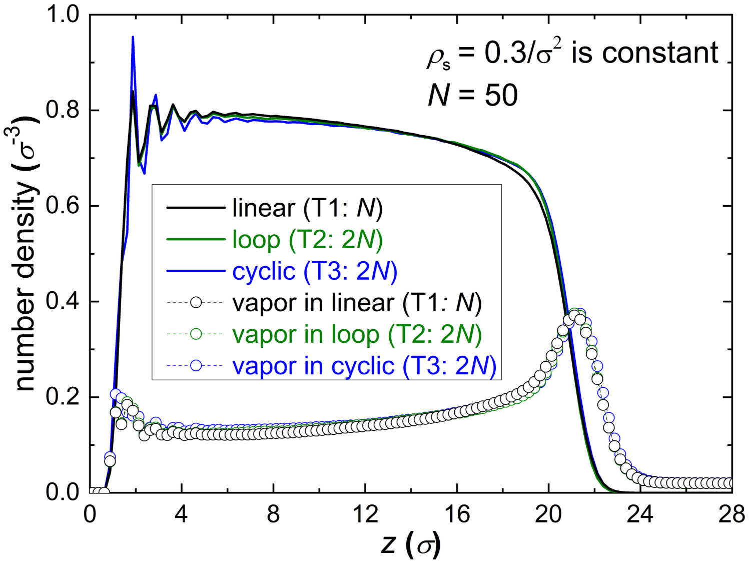

Figure S2. Density profiles of polymer brushes with different topological variations, and related density profiles of solvent molecules in corresponding polymer brushes. Here, the swollen polymer brushes are immersed in vapor phase where exists a coexistence of a solvent and a vapor phases. The solid lines between connecting circles are guides to eyes and the grafting densities of these brushes have been clarified by the definition shown in **Figure 1** of main text. The simulation parameters are chosen as $\mu = -3.5\varepsilon$, $\varepsilon_{pp} = \varepsilon_{ss} = \varepsilon_{ps} = 1.0\varepsilon$ and $\rho_s = 0.3\sigma^{-2}$. Here the chain length parameter N = 50. We see that the general properties of density profiles do not change.

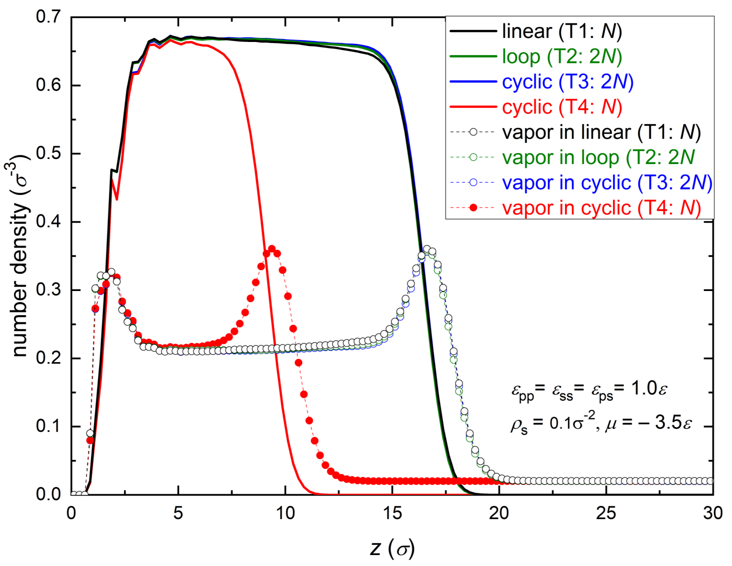

Figure S3. Density profiles of polymer brushes with different topological variations, and related density profiles of solvent molecules in corresponding polymer brushes. Here, the swollen polymer brushes are immersed in vapor phase where exists a coexistence of a solvent and a vapor phases. The solid lines between connecting circles are guides to eyes and the grafting densities of these brushes have been clarified by the definition shown in **Figure 1** of main text. The simulation parameters are chosen as $\mu = -3.5\varepsilon$, $\varepsilon_{pp} = \varepsilon_{ss} = \varepsilon_{ps} = 1.0\varepsilon$ and $\rho_s = 0.1\sigma^{-2}$.

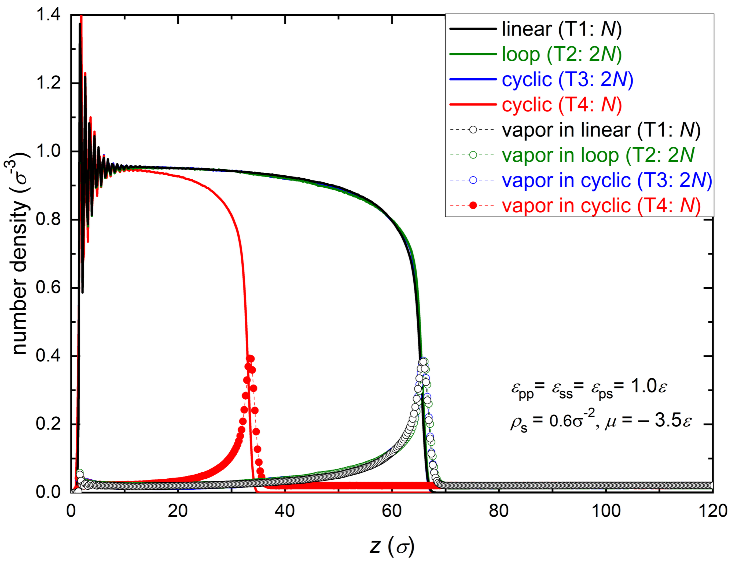

Figure S4. Density profiles of polymer brushes with different topological variations, and related density profiles of solvent molecules in corresponding polymer brushes. Here, the swollen polymer brushes are immersed in vapor phase where exists a coexistence of a solvent and a vapor phases. The solid lines between connecting circles are guides to eyes

and the grafting densities of these brushes have been clarified by the definition shown in **Figure 1** of main text. The simulation parameters are chosen as $\mu = -3.5\varepsilon$, $\varepsilon_{pp} = \varepsilon_{ss} = \varepsilon_{ps} = 1.0\varepsilon$ and $\rho_s = 0.6\sigma^{-2}$.

Section C: The effect of anchor number on the swelling properties of cyclic brushes

In **Figure S5**, the two cyclic brushes are with the same number of monomers in one chain, and they are with the same grafting density which is defined as the number of brush chains per unit area. The left T3 topology are primarily investigated in our study, the right T5 topology was considered previously by Galata and Kröger **[8]**. From the results shown in **Figure S6** and **Figure S7**, we see that the effect of anchor number on the swelling properties of cyclic brushes, is negligible in our simulations.

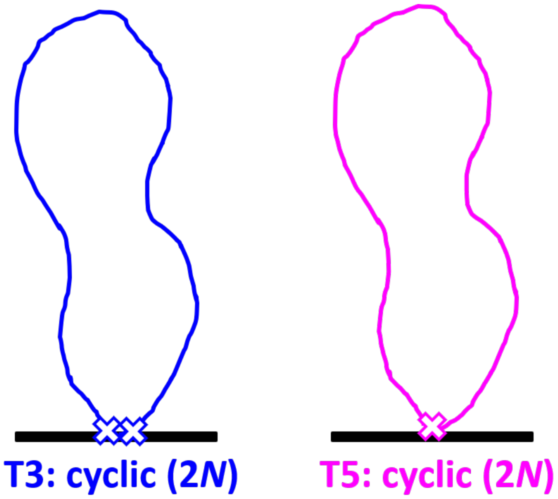

Figure S5. Cyclic brushes with different number of anchors are often studied in experiments and simulation. In the T3 topology brushes, there are two anchors at the root of one brush chain. In the T5 topology, there is one anchor at the root of one brush chain. The T3 and T5 cyclic brushes are with the same number of monomers in one chain, and they are with the same grafting density which is defined as the number of brush chains per unit area.

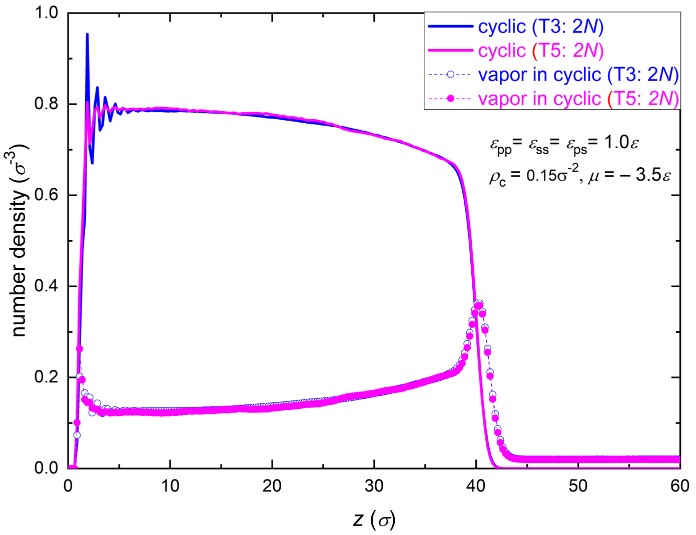

Figure S6. Density profiles of polymer brushes with T3 and T5 topologies, and related density profiles of solvent molecules in corresponding polymer brushes. Here, the swollen polymer brushes are immersed in vapor phase where exists a coexistence of a solvent and a vapor phases. The solid lines between connecting circles are guides to eyes and the grafting densities of these brushes have been clarified by the definition shown in **Figure S5**. The simulation parameters are chosen as $\mu = -3.5\varepsilon$, $\varepsilon_{pp} = \varepsilon_{ss} = \varepsilon_{ps} = 1.0\varepsilon$ and $\rho_c = 0.15\sigma^{-2}$.

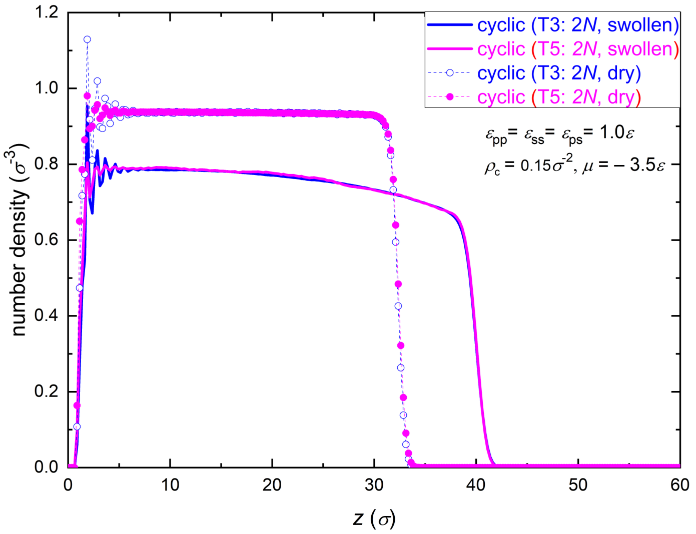

Figure S7. Density profiles of polymer brushes with T3 and T5 topologies at dry and swollen states. Here, the swollen polymer brushes are immersed in vapor phase where exists a coexistence of a solvent and a vapor phases. The solid lines between connecting circles are guides to eyes and the grafting densities of these brushes have been clarified by the definition shown in **Figure S5**. The simulation parameters are chosen as $\mu = -3.5\varepsilon$, $\varepsilon_{pp} = \varepsilon_{ss} = \varepsilon_{ps} = 1.0\varepsilon$ and $\rho_c = 0.15\sigma^{-2}$.

Section D

In this section, we show simulation data for the density profiles when the simulation parameters are chosen as $\mu = -3.5\varepsilon$, $\varepsilon_{pp} = \varepsilon_{ss} = \varepsilon_{ps} = 1.0\varepsilon$ and grafting density $\rho_s = 0.1–0.6\sigma^{-2}$. The grafting densities and topologies of polymer brushes have been clarified by the definition shown in **Figure 1** of main text.

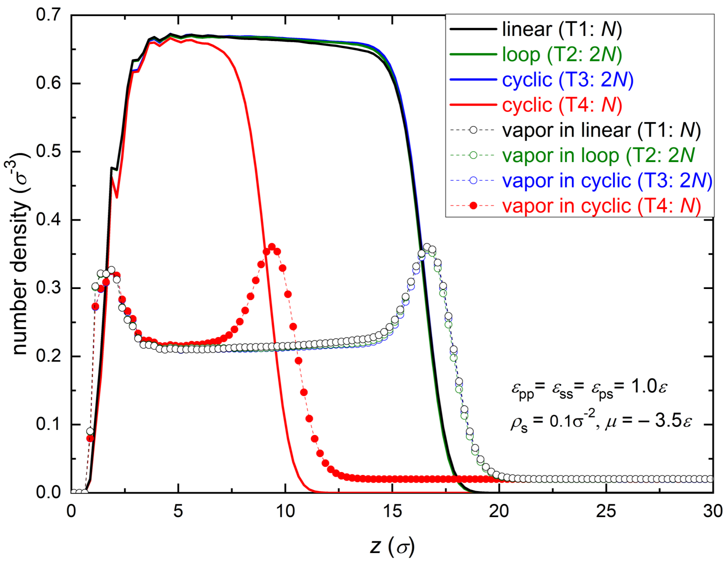

Figure S8. Density profiles of polymer brushes with different topological variations, and related density profiles of solvent molecules in corresponding polymer brushes. Here, the swollen polymer brushes are immersed in vapor phase where exists a coexistence of a solvent and a vapor phases. The solid lines between connecting circles are guides to eyes and the grafting densities of these brushes have been clarified by the definition shown in **Figure 1** of main text. The simulation parameters are chosen as $\mu = -3.5\varepsilon$, $\varepsilon_{pp} = \varepsilon_{ss} = \varepsilon_{ps} = 1.0\varepsilon$ and $\rho_s = 0.1\sigma^{-2}$.

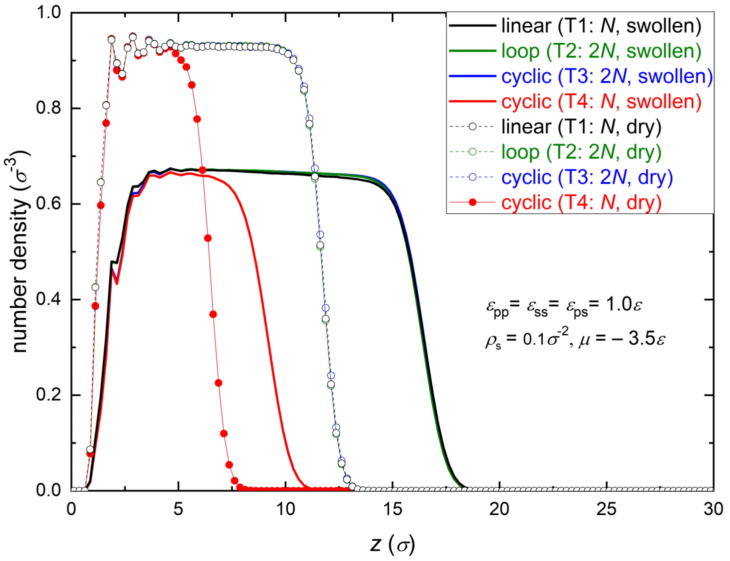

Figure S9. Density profiles of polymer brushes with different topological variations at dry and swollen states. Here, the swollen polymer brushes are immersed in vapor phase where exists a coexistence of a solvent and a vapor phases. The solid lines between connecting circles are guides to eyes and the grafting densities of these brushes have been clarified by the definition shown in **Figure 1** of main text. The simulation parameters are chosen as $\mu = -3.5\varepsilon$, $\varepsilon_{pp} = \varepsilon_{ss} = \varepsilon_{ps} = 1.0\varepsilon$ and $\rho_s = 0.1\sigma^{-2}$.

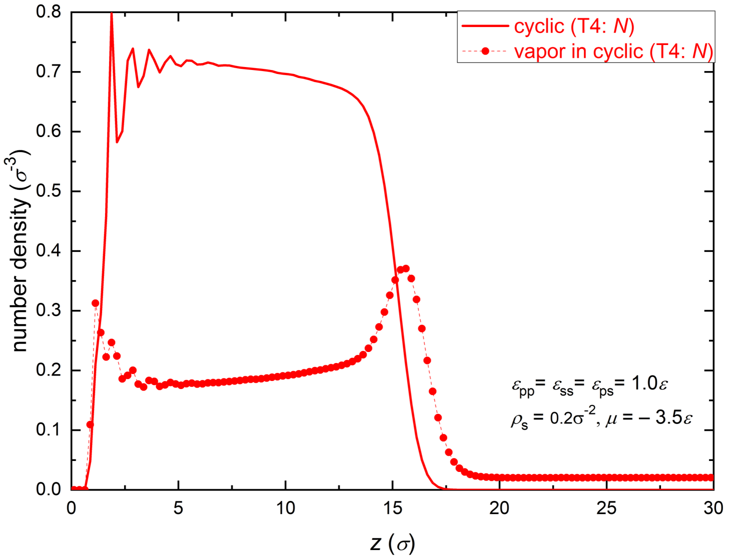

Figure S10. Density profile of cyclic polymer brushes, and related density profile of solvent molecules in polymer brushes. Here, the swollen polymer brushes are immersed in vapor phase where exists a coexistence of a solvent and a vapor phases. The solid lines between connecting circles are guides to eyes and the grafting densities of these brushes have been clarified by the definition shown in **Figure 1** of main text. The simulation parameters are chosen as $\mu = -3.5\varepsilon$, $\varepsilon_{pp} = \varepsilon_{ss} = \varepsilon_{ps} = 1.0\varepsilon$ and $\rho_s = 0.2\sigma^{-2}$.

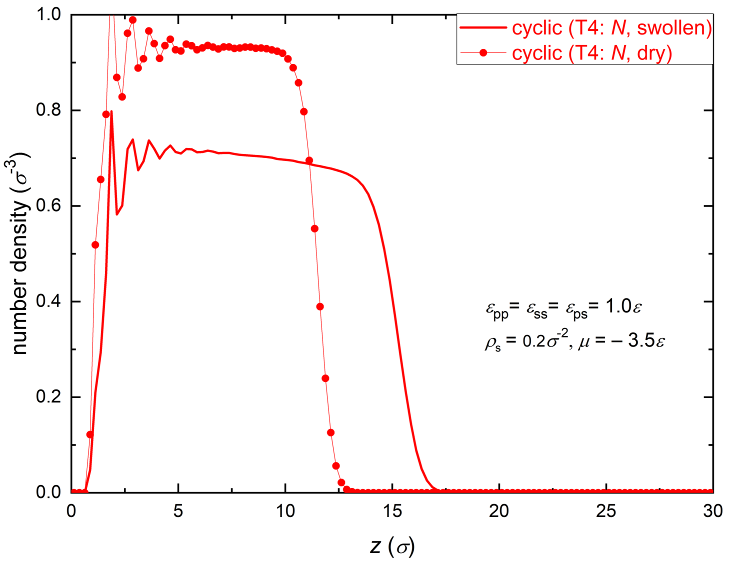

Figure S11. Density profile of cyclic polymer brushes at dry and swollen states. Here, the swollen polymer brushes are immersed in vapor phase where exists a coexistence of a solvent and a vapor phases. The solid lines between connecting circles are guides to eyes and the grafting densities of these brushes have been clarified by the definition shown in **Figure 1** of main text. The simulation parameters are chosen as $\mu = -3.5\varepsilon$, $\varepsilon_{pp} = \varepsilon_{ss} = \varepsilon_{ps} = 1.0\varepsilon$ and $\rho_s = 0.2\sigma^{-2}$.

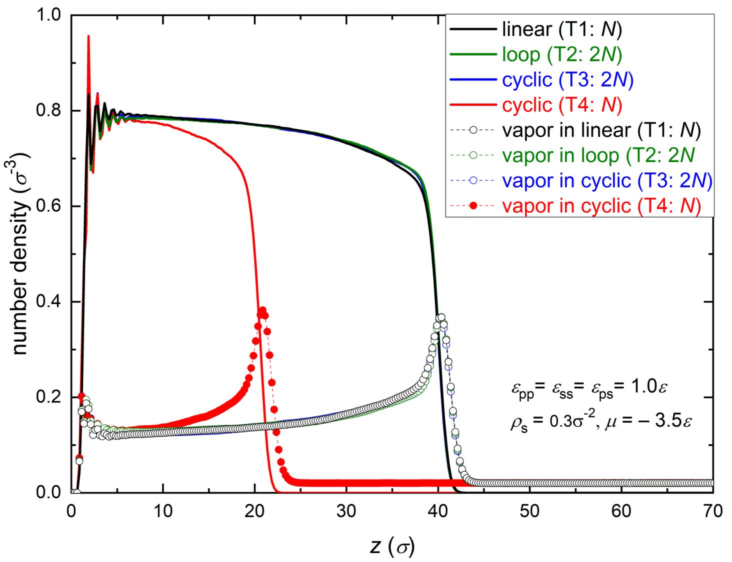

Figure S12. Density profiles of polymer brushes with different topological variations, and related density profiles of solvent molecules in corresponding polymer brushes. Here, the swollen polymer brushes are immersed in vapor phase where exists a coexistence of a solvent and a vapor phases. The solid lines between connecting circles are guides to eyes and the grafting densities of these brushes have been clarified by the definition shown in **Figure 1** of main text. The simulation parameters are chosen as $\mu = -3.5\varepsilon$, $\varepsilon_{pp} = \varepsilon_{ss} = \varepsilon_{ps} = 1.0\varepsilon$ and $\rho_s = 0.3\sigma^{-2}$.

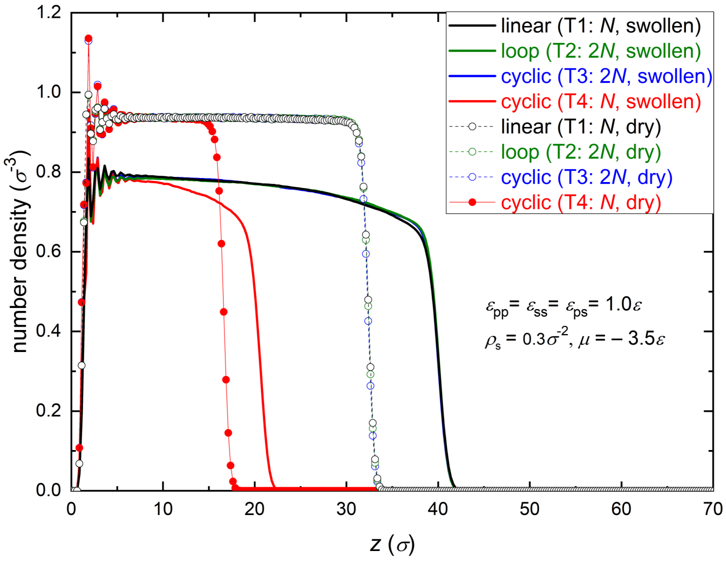

Figure S13. Density profiles of polymer brushes with different topological variations at dry and swollen states. Here, the swollen polymer brushes are immersed in vapor phase where exists a coexistence of a solvent and a vapor phases. The solid lines between connecting circles are guides to eyes and the grafting densities of these brushes have been clarified by the definition shown in **Figure 1** of main text. The simulation parameters are chosen as $\mu = -3.5\varepsilon$, $\varepsilon_{pp} = \varepsilon_{ss} = \varepsilon_{ps} = 1.0\varepsilon$ and $\rho_s = 0.3\sigma^{-2}$.

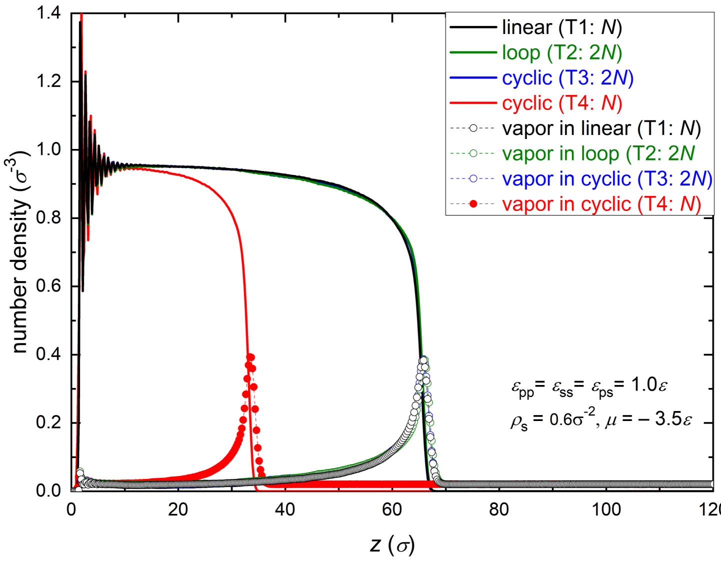

Figure S14. Density profiles of polymer brushes with different topological variations, and related density profiles of solvent molecules in corresponding polymer brushes. Here, the swollen polymer brushes are immersed in vapor phase where exists a coexistence of a solvent and a vapor phases. The solid lines between connecting circles are guides to eyes and the grafting densities of these brushes have been clarified by the definition shown in **Figure 1** of main text. The

simulation parameters are chosen as $\mu = -3.5\varepsilon$, $\varepsilon_{pp} = \varepsilon_{ss} = \varepsilon_{ps} = 1.0\varepsilon$ and $\rho_s = 0.6\sigma^{-2}$.

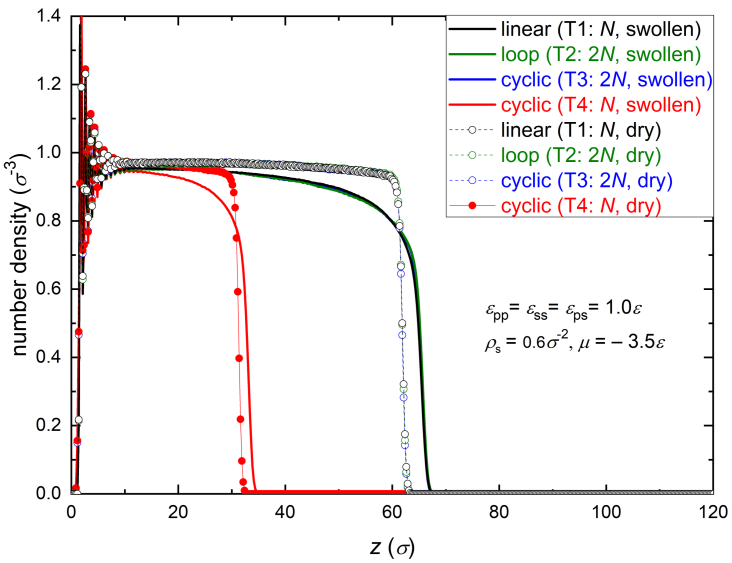

Figure S15. Density profiles of polymer brushes with different topological variations at dry and swollen states. Here, the swollen polymer brushes are immersed in vapor phase where exists a coexistence of a solvent and a vapor phases. The solid lines between connecting circles are guides to eyes and the grafting densities of these brushes have been clarified by the definition shown in **Figure 1** of main text. The simulation parameters are chosen as $\mu = -3.5\varepsilon$, $\varepsilon_{pp} = \varepsilon_{ss} = \varepsilon_{ps} = 1.0\varepsilon$ and $\rho_s = 0.6\sigma^{-2}$.

Section E

In this section, we show simulation data for the density profiles when the simulation parameters are chosen as $\mu = -3.5\varepsilon$, $\varepsilon_{pp} = \varepsilon_{ss} = 1.0\varepsilon$ with $\varepsilon_{ps} = 1.3\varepsilon$ and grafting density $\rho_s = 0.1–0.6\sigma^{-2}$. The grafting densities and topologies of polymer brushes have been clarified by the definition shown in **Figure 1** of main text.

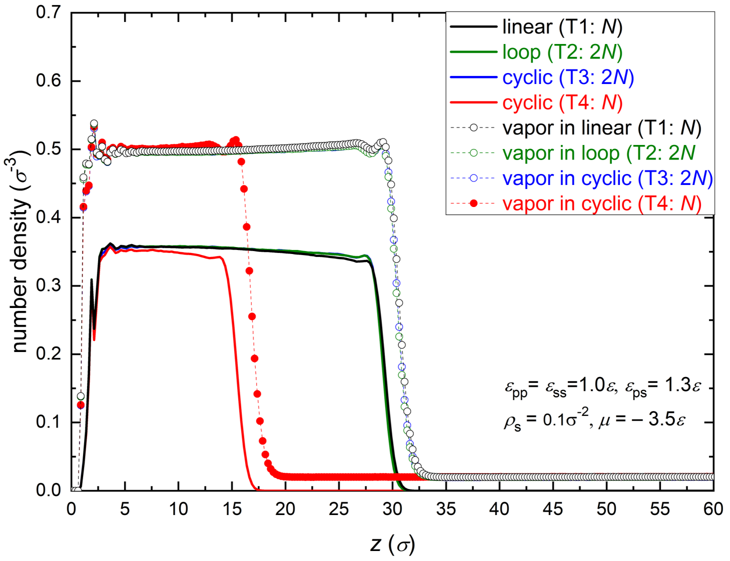

Figure S16. Density profiles of polymer brushes with different topological variations, and related density profiles of solvent molecules in corresponding polymer brushes. Here, the swollen polymer brushes are immersed in vapor phase where exists a coexistence of a solvent and a vapor phases. The solid lines between connecting circles are guides to eyes and the grafting densities of these brushes have been clarified by the definition shown in **Figure 1** of main text. The simulation parameters are chosen as $\mu = -3.5\varepsilon$, $\varepsilon_{pp} = \varepsilon_{ss} = 1.0\varepsilon$ with $\varepsilon_{ps} = 1.3\varepsilon$ and $\rho_s = 0.1\sigma^{-2}$.

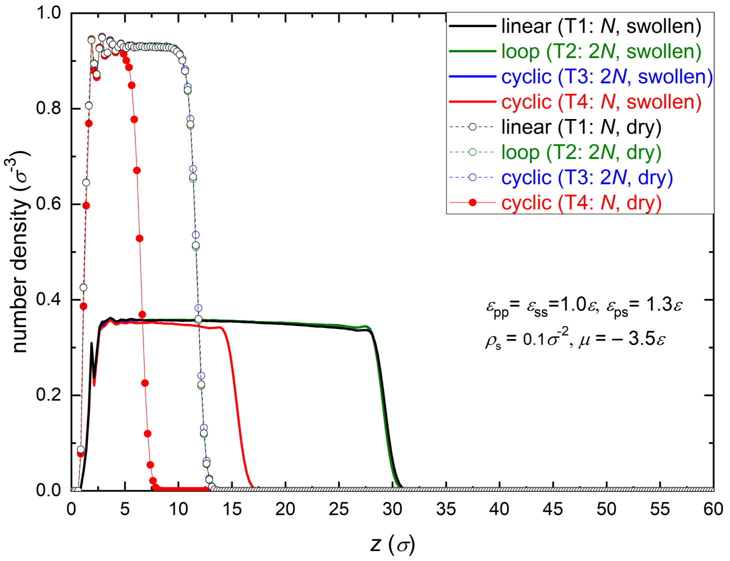

Figure S17. Density profiles of polymer brushes with different topological variations at dry and swollen states. Here, the swollen polymer brushes are immersed in vapor phase where exists a coexistence of a solvent and a vapor phases. The solid lines between connecting circles are guides to eyes and the grafting densities of these brushes have been clarified by the definition shown in **Figure 1** of main text. The simulation parameters are chosen as $\mu = -3.5\varepsilon$, $\varepsilon_{pp} = \varepsilon_{ss} = 1.0\varepsilon$ with $\varepsilon_{ps} = 1.3\varepsilon$ and $\rho_s = 0.1\sigma^{-2}$.

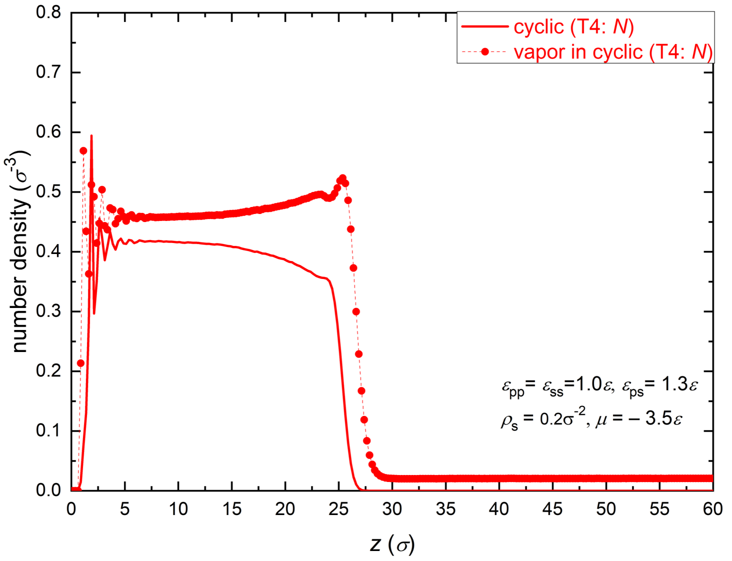

Figure S18. Density profile of cyclic polymer brushes, and related density profile of solvent molecules in polymer brushes. Here, the swollen polymer brushes are immersed in vapor phase where exists a coexistence of a solvent and a vapor phases. The solid lines between connecting circles are guides to eyes and the grafting densities of these brushes have been clarified by the definition shown in **Figure 1** of main text. The simulation parameters are chosen as $\mu = -3.5\varepsilon$, $\varepsilon_{pp} = \varepsilon_{ss} = 1.0\varepsilon$ with $\varepsilon_{ps} = 1.3\varepsilon$ and $\rho_s = 0.2\sigma^{-2}$.

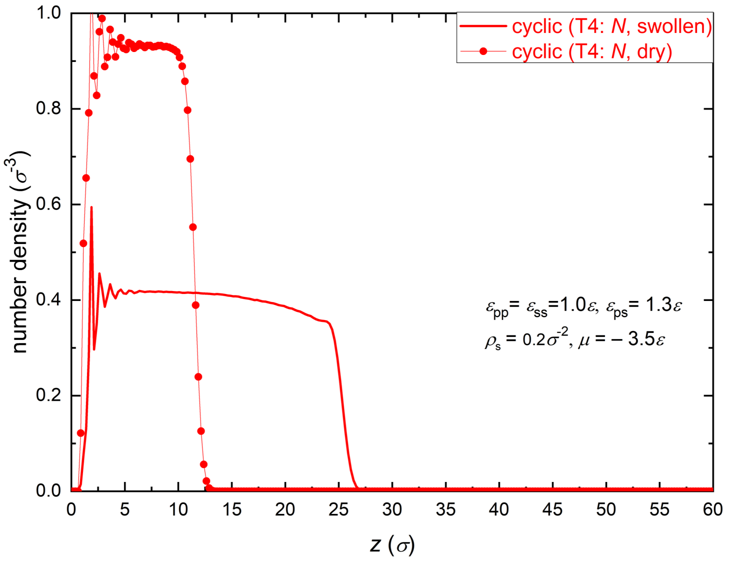

Figure S19. Density profile of cyclic polymer brushes at dry and swollen states. Here, the swollen polymer brushes are immersed in vapor phase where exists a coexistence of a solvent and a vapor phases. The solid lines between connecting circles are guides to eyes and the grafting densities of these brushes have been clarified by the definition shown in **Figure 1** of main text. The simulation parameters are chosen as $\mu = -3.5\varepsilon$, $\varepsilon_{pp} = \varepsilon_{ss} = 1.0\varepsilon$ with $\varepsilon_{ps} = 1.3\varepsilon$ and $\rho_s = 0.2\sigma^{-2}$.

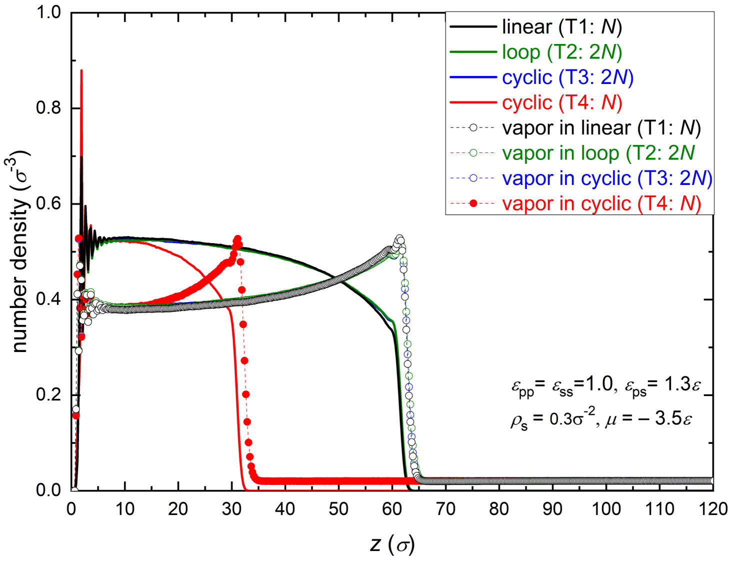

Figure S20. Density profiles of polymer brushes with different topological variations, and related density profiles of solvent molecules in corresponding polymer brushes. Here, the swollen polymer brushes are immersed in vapor phase where exists a coexistence of a solvent and a vapor phases. The solid lines between connecting circles are guides to eyes and the grafting densities of these brushes have been clarified by the definition shown in **Figure 1** of main text. The simulation parameters are chosen as $\mu = -3.5\varepsilon$, $\varepsilon_{pp} = \varepsilon_{ss} = 1.0\varepsilon$ with $\varepsilon_{ps} = 1.3\varepsilon$ and $\rho_s = 0.3\sigma^{-2}$.

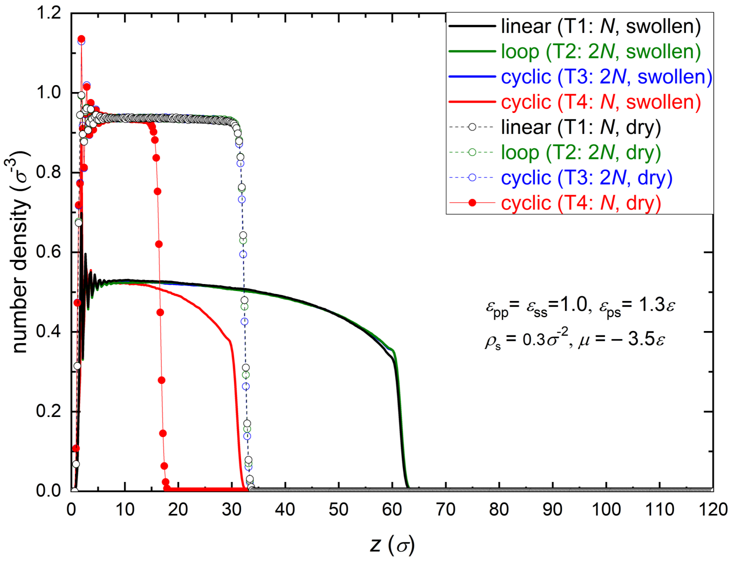

Figure S21. Density profiles of polymer brushes with different topological variations at dry and swollen states. Here, the swollen polymer brushes are immersed in vapor phase where exists a coexistence of a solvent and a vapor phases. The solid lines between connecting circles are guides to eyes and the grafting densities of these brushes have been clarified by the definition shown in **Figure 1** of main text. The simulation parameters are chosen as $\mu = -3.5\varepsilon$, $\varepsilon_{pp} = \varepsilon_{ss} =$

1.0ε with $\varepsilon_{ps} = 1.3\varepsilon$ and $\rho_s = 0.3\sigma^{-2}$.

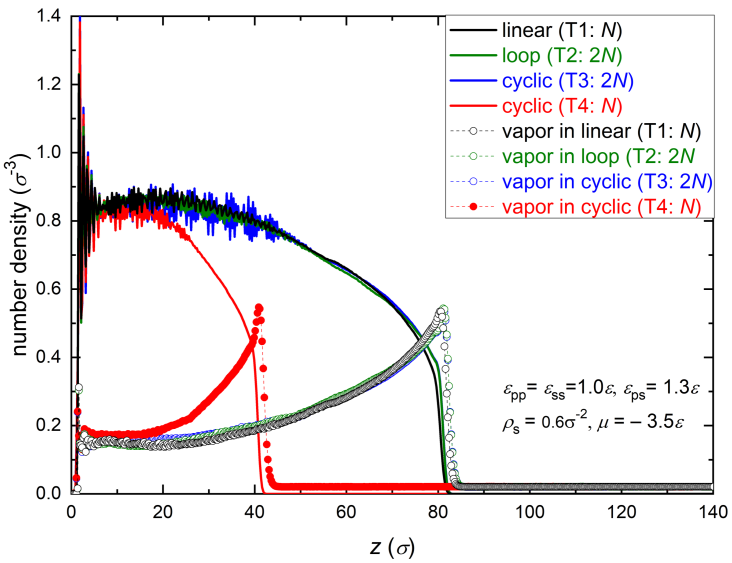

Figure S22. Density profiles of polymer brushes with different topological variations, and related density profiles of solvent molecules in corresponding polymer brushes. Here, the swollen polymer brushes are immersed in vapor phase where exists a coexistence of a solvent and a vapor phases. The solid lines between connecting circles are guides to eyes and the grafting densities of these brushes have been clarified by the definition shown in **Figure 1** of main text. The simulation parameters are chosen as $\mu = -3.5\varepsilon$, $\varepsilon_{pp} = \varepsilon_{ss} = 1.0\varepsilon$ with $\varepsilon_{ps} = 1.3\varepsilon$ and $\rho_s = 0.6\sigma^{-2}$.

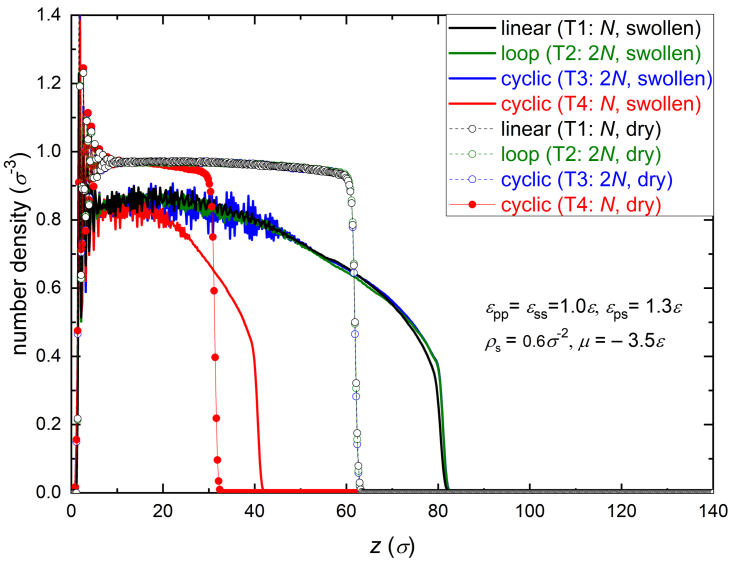

Figure S23. Density profiles of polymer brushes with different topological variations at dry and swollen states. Here, the swollen polymer brushes are immersed in vapor phase where exists a coexistence of a solvent and a vapor phases.

The solid lines between connecting circles are guides to eyes and the grafting densities of these brushes have been clarified by the definition shown in **Figure 1** of main text. The simulation parameters are chosen as $\mu = -3.5\varepsilon$, $\varepsilon_{pp} = \varepsilon_{ss} = 1.0\varepsilon$ with $\varepsilon_{ps} = 1.3\varepsilon$ and $\rho_s = 0.6\sigma^{-2}$.

Section F: The derivation of Equation (1) in the main text

In this section we briefly recapitulate the derivation of **Equation (1)** in the main text. The reader can also refer our previous work **[1]** for more details. The introduction of chain elasticity and the absence of translational entropy of brush chains lead to the free-energy expression for uncharged polymer brushes as F_{mix} in units of k_BT:

$$\frac{F_{mix}}{k_BT} = n_p\left(\frac{3h^2}{2N}\right) + n_s \ln\left(\phi_s\right) + \chi n_s \phi_p \tag{6}$$

with N being the number of monomers in a brush chain, n_p and n_s being respectively the number of brush chains and the number of solvent molecules in the brush phase, ϕ_p and $\phi_s = 1 - \phi_p$ being respectively the volume fraction of monomers and the volume fraction of solvent molecules in the brush phase. In **Equation (6)**, the swollen height of the polymer brushes is denoted as h, the Flory-Huggins interaction parameter **[9, 10]** between monomer and solvent is denoted as χ. The first term of **Equation (6)** describes the chain elasticity, which assumes that the brush chain is close to an ideal Gaussian chain if it is very long. The second term of **Equation (6)** is the mixing entropy of solvent molecules in the brush phase and the third term takes care of the non-ideal mixing such as the enthalpic interaction between monomers and solvent molecules.

The Alexander−de Gennes approach **[11, 12]** is an approximation at the mean-field level and it is primarily limited to brushes with the same chain length (i.e., monodisperse), as it relies on the assumption that every polymer chain occupies the same volume. This results in the height of the brush becomes directly proportional to the number of particles per unit area. Per polymer chain, this is approximated as

$$h = \frac{N\rho_s}{\phi_p} \tag{7}$$

As a result, the elasticity term can also be expressed in the following form

$$n_p\left(\frac{3N}{2}\right)\left(\frac{\rho_s}{\phi_p}\right)^2 \tag{8}$$

Taking the derivative of this elasticity-adjusted free-energy expression with respect to the amount of absorbed solvent yields the chemical potential for the solvent within the brush as μ_{in}:

$$\frac{\mu_{in}}{k_BT} = \ln\left(1-\phi_p\right) + \phi_p + \chi\phi_p^2 + \frac{3\rho_s^2}{\phi_p} \tag{9}$$

At the chemical equilibrium, the chemical potentials for the solvent inside the brush and for

the solvent vapor phase are equal. Ideally, the chemical potential of the bulk vapor is given by $\mu_{\rm out}$:

$$\frac{\mu_{out}}{k_B T} = \ln\left(\frac{p}{p_{sat}}\right) \tag{10}$$

Hence, the equilibrium absorption behavior of the brush is determined by $\mu_{\rm in} = \mu_{\rm out}$, which is given by **Equation (1)** in the main text. Here, we quote it for convenience as the following equation.

$$\ln\left(\frac{p}{p_{sat}}\right) = \ln\left(1-\phi_p\right) + \phi_p + \chi\phi_p^2 + \frac{3\rho_s^2}{\phi_p} \tag{11}$$

It is worth pointing out that the real brush chain always has a finite chain length in experiments. Thus, the real brush chain cannot be completely viewed as an ideal chain as the Alexander-de Gennes approximation **[11, 12]** assumes. This issue becomes important when the grafting density is very high where the brush chain is strongly stretched. An elaborated modification for the chain elasticity based on the inverse Langevin equation **[13]** may be necessary for this situation, but this lies beyond the primary goal of the present study and we will consider it in detail in future.

Section G: The fit performance of the Flory-Huggins theory

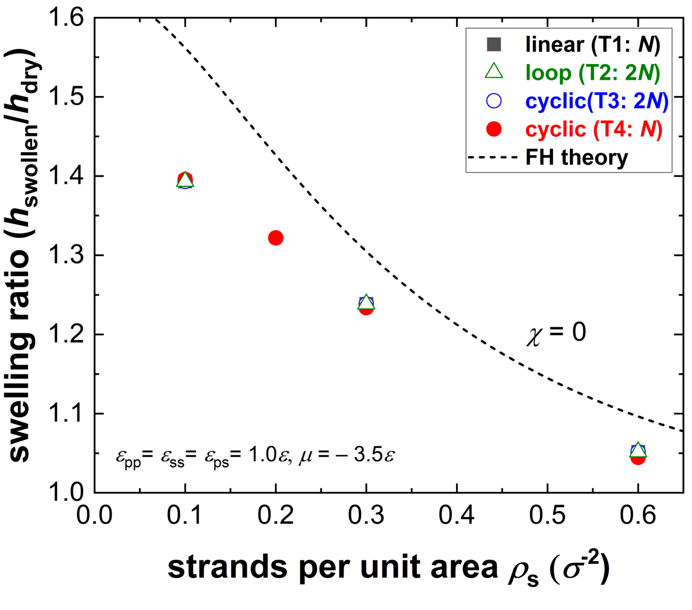

Figure S24. The fit performance of the Flory-Huggins theory for the simulation case: $\mu = -3.5\varepsilon$ with $\varepsilon_{\rm pp} = \varepsilon_{\rm ss} = \varepsilon_{\rm ps} = 1.0\varepsilon$. The fit value for the model parameter χ is fixed at $\chi = -\varepsilon_{\rm ps} + (\varepsilon_{\rm pp} + \varepsilon_{\rm ss})/2 = 0$.

In this section, we show the fit performance of the Flory-Huggins theory to our simulation data. **In this study, the brush thickness is extracted from the location of the inflection point in the polymer density profile.** We further use the following equation to connect the swelling ratio with the Flory-Huggins theory **[1]**, i.e., **Equation (11)**.

$$\frac{h_{swollen}}{h_{dry}} = \frac{1}{1-\phi_s} = \frac{1}{\phi_p} \quad (12)$$

here h_{swollen} and h_{dry} are respectively the swollen and dry thickness of the same polymer brushes.

In the **Figure S24** and **Figure S25**, we show the fit performance of the Flory-Huggins theory for the simulation cases in this study under the Alexander-de Gennes approximation **[11, 12]** for brush chain elasticity.

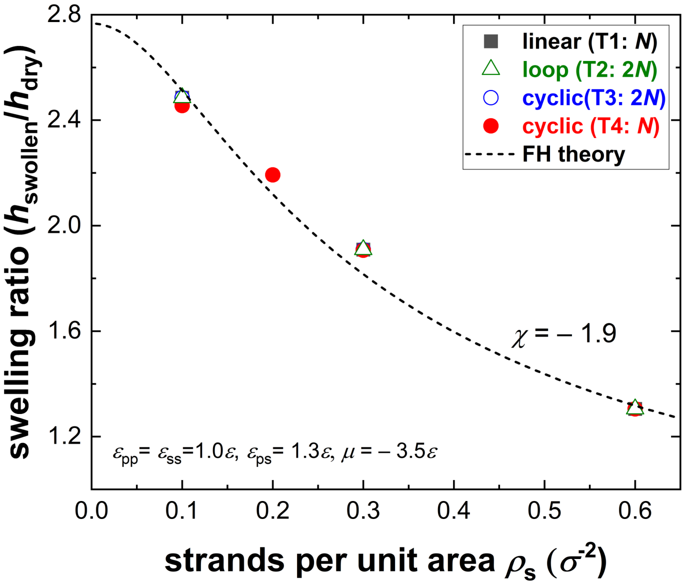

Figure S25. The fit performance of the Flory-Huggins theory for the simulation case: $\mu = -3.5\varepsilon$ and $\varepsilon_{pp} = \varepsilon_{ss} = 1.0\varepsilon$ with $\varepsilon_{ps} = 1.3\varepsilon$. The fit value for the model parameter $\chi = -1.9$, is determined based on a mapping method reported in our previous work **[14]**.

In **Figure S26** and **Figure S27**, we show the prediction of the Flory-Huggins theory for the density profiles of brushes. We note that the Flory-Huggins theory is a mean-field theory, i.e., **Equation (11)**, which predicts a box-like density distribution for monomer density. From **Figure S26** and **Figure S27**, we see that the mean-field prediction for the value of monomer density in brush phase is close to its average simulation value.

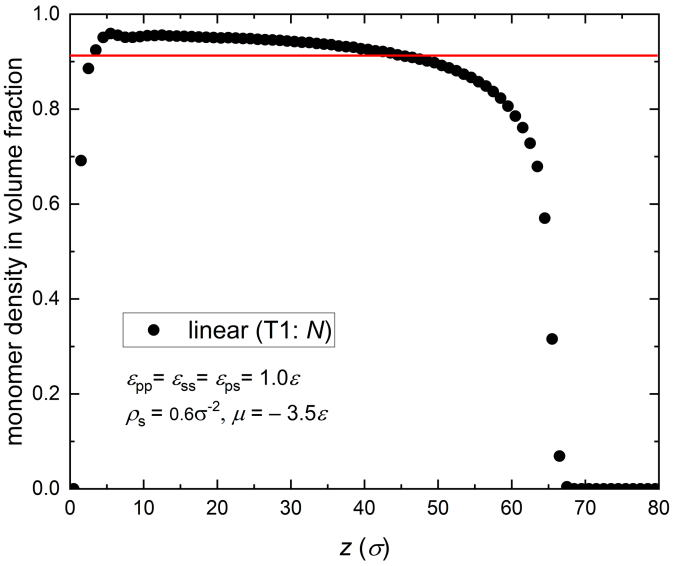

Figure S26. Density profiles of polymer brushes in volume fraction for the linear topology brushes for the simulation case of $\mu = -3.5\varepsilon$ with $\varepsilon_{pp} = \varepsilon_{ss} = \varepsilon_{ps} = 1.0\varepsilon$, and the grafting density of the linear brushes is $\rho_s = 0.6\sigma^{-2}$. The red line in the figure represents the prediction by the Flory-Huggins theory that is a mean-field value, which is based on data fit of **Figure S24.**

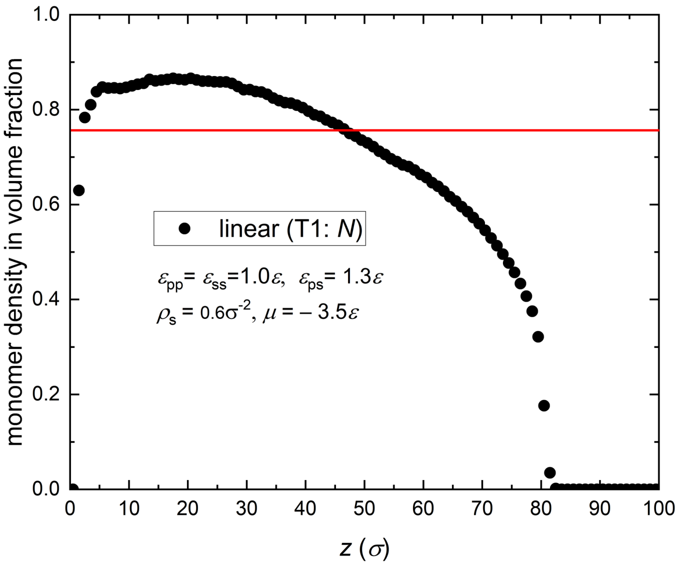

Figure S27. Density profiles of polymer brushes in volume fraction for the linear topology brushes for the simulation case of $\mu = -3.5\varepsilon$ and $\varepsilon_{pp} = \varepsilon_{ss} = 1.0\varepsilon$ with $\varepsilon_{ps} = 1.3\varepsilon$, and the grafting density of the linear brushes is $\rho_s = 0.6\sigma^{-2}$. The red line in the figure represents the prediction by the Flory-Huggins theory that is a mean-field value, which is based on data fit of **Figure S25**.

Supporting references

[1] G.C. Ritsema van Eck, L.B. Veldscholte, J.H.W.H. Nijkamp, S. de Beer, Sorption Characteristics of Polymer Brushes in Equilibrium with Solvent Vapors, Macromolecules, 53 (2020) 8428-8437.

[2] D. Frenkel, B. Smit, Understanding Molecular Simulation: From Algorithms to Applications (3rd Edition), Academic Press, San Diego, USA, 2023.

[3] S. Plimpton, Fast Parallel Algorithms for Short-Range Molecular Dynamics, Journal of Computational Physics, 117 (1995) 1-19.

[4] K. Kremer, G.S. Grest, Dynamics of entangled linear polymer melts: A molecular-dynamics simulation, The Journal of Chemical Physics, 92 (1990) 5057-5086.

[5] E. Braun, S.M. Moosavi, B. Smit, Anomalous Effects of Velocity Rescaling Algorithms: The Flying Ice Cube Effect Revisited, Journal of Chemical Theory and Computation, 14 (2018) 5262-5272.

[6] M. Tuckerman, B.J. Berne, G.J. Martyna, Reversible multiple time scale molecular dynamics, The Journal of Chemical Physics, 97 (1992) 1990-2001.

[7] L.B. Veldscholte, MDBrushGenerator, DOI 10.5281/zenodo.3945235(2020).

[8] A.A. Galata, M. Kröger, Topological Biopassive Brushes. From Linear to Cyclic, from Atomistic to Coarse-Grained Poly(2-ethyl-2-oxazoline), Macromolecules, 57 (2024) 5313-5329.

[9] M.L. Huggins, Solutions of Long Chain Compounds, The Journal of Chemical Physics, 9 (1941) 440-440.

[10] P.J. Flory, Thermodynamics of High Polymer Solutions, The Journal of Chemical Physics, 10 (1942) 51-61.

[11] P.G. De Gennes, Scaling theory of polymer adsorption, Journal de Physique, 37 (1976) 1445-1452.

[12] S. Alexander, Adsorption of chain molecules with a polar head a scaling description, Journal de Physique, 38 (1977) 983-987.

[13] M. Kröger, Simple, admissible, and accurate approximants of the inverse Langevin and Brillouin functions, relevant for strong polymer deformations and flows, Journal of Non-Newtonian Fluid Mechanics, 223 (2015) 77-87.

[14] L.A. Smook, G.C. Ritsema van Eck, S. de Beer, Friends, Foes, and Favorites: Relative Interactions Determine How Polymer Brushes Absorb Vapors of Binary Solvents, Macromolecules, 53 (2020) 10898-10906.
